# The impact of population aging on SME digital transformation: Evidence from China

**DOI:** 10.1371/journal.pone.0300660

**Published:** 2024-05-16

**Authors:** Xueyan Xie, Xiaohui Xu

**Affiliations:** Beijing Technology and Business University, China, Beijing; Public Library of Science, UNITED KINGDOM

## Abstract

Many societies around the world are rapidly aging and this has implications for social and economic development. We collect data on NEEQ-listed enterprises from 2010 to 2021 in China and empirically test the effect of population aging on the digital transformation of small and medium-sized enterprises (SMEs). The findings show that population aging has a significant positive impact on SME digital transformation, and private enterprises and enterprises in eastern regions of China tend to benefit more than other regions. The mechanism studies find that population aging positively impacts SME digital transformation by increasing labor costs, facilitating human capital accumulation, and raising savings rates. Furthermore, the threshold effect analyses find that the marginal promotion effect of population aging will weaken with greater aging and will strengthen with a higher level of marketization. Finally, we provide policy recommendations for promoting digital transformation in SMEs against the background of population aging.

## 1. Introduction

Population aging refers to changes in the age structure of the population and an increase in the proportion of the elderly in the total population. It is a global social phenomenon and a reality that many countries must face. According to the data from the National Bureau of Statistics, the total population of China at the end of 2022 decreased by 850,000 compared with the end of the previous year, and the natural population growth rate was -0.60%, marking the first negative population growth rate in China in more than 60 years. Meanwhile, the elderly population aged 65 and above exceeded 210 million, accounting for 14.9% of the total population. According to international standards, China has entered the stage of moderate aging. Studies show that population aging has significant effects on social and economic development. At the macro level, population aging can promote the application of automation technology [1], enhance technological innovation [2, 3], and drive industrial structure upgrading [4] and economic growth [5]. At the micro level, population aging can affect the allocation of household financial assets [6], enterprises’ debt financing decisions [7], and investment behaviors [8].

At the same time, SMEs have become an important part of the Chinese economy by playing an essential role in expanding employment, narrowing income gaps, promoting market competition, and driving innovation. With the development of the digital economy in recent years, SMEs are actively seeking digital transformation. Enterprise digital transformation is the deep integration of digital technology and enterprise strategies, which can trigger the digital evolution of resources and capabilities, business models, business processes, products and services, and business ecosystems [9]. Studies find that digital transformation is beneficial in reducing enterprise debt costs [10], alleviating financial constraints [11], improving internal and external governance environments [12, 13], enhancing performance levels [14, 15], reducing business risks [16], promoting technological innovation [17–19], and increasing total factor productivity [20, 21]. Therefore, the digital transformation of SMEs is crucial to the development of the enterprises themselves as well as the national economy, and this topic has received wide attention from governments and researchers.

What kind of impact will population aging have on SME digital transformation? There are few studies that directly explore the relationship between the two. It is well known that enterprise digital transformation based on innovation in enterprise digital technology is an investment behavior of enterprises. Relevant studies explore the impact of population aging on technological innovation and investment. For example, Liu et al. [2] find that population aging could stimulate enterprises to increase R&D investment, introduce high-tech personnel, and improve the level of technological innovation. Similarly, Baldanzi et al. [3] show that aging leads to an increase in life expectancy, aggregate savings, and demand for innovation, thereby causing higher employment in the R&D sector and faster technological progress. Xian et al. [8] document that population aging affects the investment decisions of enterprises by increasing the labor cost of manufacturing enterprises and reducing the output efficiency of unit wages. Therefore, we use data on NEEQ-listed enterprises from 2010 to 2021 in China and find that population aging has a significant positive impact on SME digital transformation. Moreover, we explore three influence mechanisms. First, population aging leads to higher labor costs, forcing enterprises to replace labor with capital and technology, which drives SMEs’ digital transformation. Second, population aging means longer life expectancy and higher return on investment in education, promoting human capital accumulation, which provides intellectual support to SMEs’ digital transformation. Third, population aging raises savings rates, alleviating financial constraints, which provides financial support for SMEs’ digital transformation. Studies on heterogeneity find that the positive impact of population aging on enterprise digital transformation is more significant for private enterprises and enterprises in eastern regions of China. Finally, we find that the impact of population aging on SME digital transformation is nonlinear. The marginal effect of population aging weakens with greater aging but strengthens with a higher level of marketization.

The theoretical contributions of our paper are as follows. First, in terms of the research perspective, we examine the impact of population aging, an indispensable macro factor, on enterprise digital transformation, thus enriching the literature on population aging and the digital transformation of enterprises. Many studies focus on the economic consequences of population aging, such as the level of technological innovation [2, 3], the upgrading of industrial structure [4], and economic growth [5], but what will be the impact on the digital transformation of enterprises, an important trend of enterprise development? Relevant studies are still lacking. Moreover, existing studies discuss the factors influencing enterprise digital transformation from multiple perspectives, such as organizational structure [22], corporate strategy [23], and policy support [24], but few studies pay attention to the impact of changes in demographics, which is an important macro factor. Second, in terms of research content, we analyze the influence paths of population aging on SME digital transformation based on the effects of labor costs, human capital, and savings rates. The literature has not yet focused on the effect of population aging on enterprise digital transformation and its mechanisms and our paper fills this gap. Third, in terms of research methodology, we adopt a textual analysis method to characterize SME digital transformation by constructing a more comprehensive keyword dictionary of digital transformation that includes 169 keywords and then counting the keyword frequency, hence providing a useful reference for measuring the degree of SMEs’ digital transformation. Compared to established studies, we not only include keywords in digital technology but also expand keywords in digital technology applications.

The remainder of this paper is organized as follows. Section 2 provides the literature review. Section 3 describes the theoretical analysis and hypothesis. Section 4 describes the variable constructions, data, and model. Section 5 presents the results for the baseline regression, robustness checks, and endogeneity tests. Section 6 explores the influence mechanisms. Section 7 tests the nonlinear impact. Section 8 draws conclusions and issues recommendations.

## 2. Literature review

With population aging becoming a global problem, scholars have conducted a series of studies on the economic impacts of population aging. At the macro level, studies find that population aging has both positive and negative impacts. On the positive side, Annabi et al. [25] construct an overlapping generations (OLG) model and find that the labor supply shortage caused by aging increases the average wage level and promotes the relative expansion of nonlabor-intensive industries. Siliverstovs et al. [26] use data from developing and developed countries from 1970–2004 and indicate that population aging has a significant positive effect on the employment share of the tertiary sector. Chu et al. [4] construct a spatial econometric SEM from the perspective of production and consumption and discover that population aging has a dynamic impact on industrial structure upgrading by promoting the substitution of capital or technology for labor and increasing per capita education years. On the negative side, Sanz and Velázquez [27] use government expenditure data from OECD countries during 1970–1997 and reveal that the increase in the proportion of elderly people would lead to an increase in public expenditure and undermine the sustainability of public finances, negatively impacting economic growth. Using state-level data from the US, Maestas et al. [28] estimate that slower labor force growth due to aging negatively affects national per capita output growth. Prettner et al. [29] propose a dynamic consumer optimization model and show that the economic decline caused by a slowdown in labor supply can be partially offset by increasing investment in higher education and health care. At the micro level, Bressan et al. [6], based on data from the Health, Aging and Retirement in Europe survey, find that people would reduce risk-taking behaviors and prefer safer investment portfolios as they become older and less healthy. Chen et al. [7] use data from 2007 to 2019 of Chinese A-share listed companies and discover that population aging reduces the level of corporate debt financing and aggravates the mismatch of financial resources. Xian et al. [8] use data from 2012 to 2020 of A-share listed manufacturing companies in China and document that population aging reduces the output efficiency of unit labor costs and prompts companies to increase foreign investment.

In recent years, with the development and application of digital technology worldwide, more studies on digital transformation have emerged. Some studies focus on the factors that influence digital transformation. At the micro level, these factors include manager characteristics [30], organizational structure [22], dynamic capabilities [31], profit expectations [32], digital strategies [23], financial asset allocation [33], and equity structure [34]. At the macro level, these factors include digital technology [35], financial technology [36], digital infrastructure construction [37], policy guarantees [24], and the business environment [38]. In addition, some studies focus on the economic effects of digital transformation. Cui et al. [16] use data from 2012 to 2021 of Chinese A-share listed companies and find that digital transformation alleviates financial distress by reducing operational risks and easing financial constraints. Manita et al. [13], based on qualitative data analysis, reveal that the application of digital technology reduces information asymmetry and improves the internal governance environment of enterprises. Zhai et al. [14] use data from 2009 to 2019 of A-share listed companies in China and conduct empirical studies which show that digital transformation reduces operating costs, improves operational efficiency, and thereby enhances performance levels. Blichfeldt and Faullant [18] use European Manufacturing Survey (EMS) data and find that companies with a high level of digital technology implementation (in both breadth and depth) could introduce more radical product and service innovations. Wen et al. [19], based on data from Chinese A-share listed manufacturing companies from 2007 to 2019, discover that digital transformation increases companies’ investment in innovation activities and promotes technological innovation. Yu et al. [21] select data from listed companies from 2015 to 2021 as a research sample and show that enterprises that use digital technology can accelerate internal knowledge spillovers and improve enterprise competitiveness, thereby increasing total factor productivity.

## 3. Theoretical analysis and hypothesis

### 3.1 The impact of population aging on SME digital transformation

Digital transformation is characterized by long cycles and high uncertainty, requiring low-cost funds in the long term as well as talent and technology as complements. Because of this, SMEs face greater challenges in digital transformation than large enterprises. Population aging leads to higher labor costs, human capital, and savings rates, which creates favorable conditions for enterprises to utilize capital, talent, and technology, thus promoting the digital transformation of SMEs. First, the rise in the proportion of the elderly population causes a shortage of working-age laborers, leading to an increase in labor costs and forcing enterprises to shift their factor preference from labor to capital and technology [39], thus becoming a push for SMEs’ digital transformation. Second, population aging will extend life expectancy and provide a longer time window [40] and greater incentives [41] for people to pursue higher education. These factors promote human capital accumulation, and improve the quality of the workforce, which can meet the demand of enterprises for talent and become a pull for SMEs’ digital transformation. Third, population aging may lead to a rise in the savings rate [42], which alleviates the financial constraints faced by SMEs. This is conducive to improving the affordability of digital transformation to SMEs, becoming another pull for SMEs’ digital transformation. Based on the above analysis, the following hypothesis is proposed.

Hypothesis 1: Population aging can positively impact the digital transformation of SMEs.

**Fig 1:**
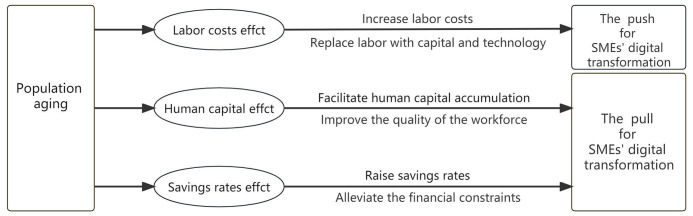
Mechanisms of population aging on the digital transformation of SMEs.

The mechanisms of population aging on the digital transformation of SMEs are displayed in Fig 1.

### 3.2 The mediating role of labor costs

Population aging is accompanied by a decline in the birth rate, which leads to an increase in the average age and a decline in the labor participation rate of working-age laborers. Therefore, as population aging deepens, the labor supply becomes relatively scarce in the factor market, labor costs increase [25], and the relative prices of production factors change. Compared with large enterprises, SMEs are more inclined to labor-intensive industries. Meanwhile, labor is the most basic and important production factor for SMEs, and a reduction in its supply and cost increases will inevitably have a greater negative influence on SMEs. To offset these negative impacts, enterprises are forced to adjust production strategies, redistribute limited funds among different factors, and replace labor with capital and technology [39]. From the experience of developed countries, countries turn to high-tech industries as the population ages to create more value with less labor. In recent years, with the development of digital technologies such as big data, artificial intelligence, and automation, the trend of using machines to fill labor shortages has become even more apparent. Acemoglu and Restrepo [1] use panel data from 126 countries during 1990–2015 and find that countries with a higher degree of aging offset the negative effects of labor reduction through large-scale industrial automation, thus minimizing the risk of economic downturns. From this, the replacement of labor by capital and technology becomes an important component of strategic adjustments as the population ages, thus promoting the digital transformation of SMEs. Hence, the following hypothesis is postulated.

Hypothesis 2: Population aging can affect SMEs by the push effect of increasing labor costs and thus positively impact the digital transformation of SMEs.

### 3.3 The mediating role of human capital accumulation

Enterprise digital transformation means the digitalization, automation, and intelligence of production, which requires highly skilled labor. For example, enterprises need to hire highly skilled engineers to install, debug, use, and maintain advanced production equipment after it has been purchased. In addition, technological innovation promotes enterprise digital transformation, which itself is a technological innovation activity [43]. Technological research requires advanced abilities in thinking, learning, cooperation, psychological qualities, and innovation in workers. Therefore, enterprise digital transformation requires high-level human capital. At the same time, population aging extends life expectancy. Since it increases the time that people can use to enrich themselves, more young people will choose to postpone entering the labor market to receive more education [40]. It also increases the expected rate of return on investment of education, which incentivizes people to improve the qualities of family members, thus increasing human capital investment [41]. In summary, population aging facilitates human capital accumulation and knowledge spillover, which provides the necessary intellectual support for SMEs to implement digital transformation. Consequently, the following hypothesis is proposed.

Hypothesis 3: Population aging can affect SMEs by the pull effect of facilitating human capital accumulation and thus positively impact the digital transformation of SMEs.

### 3.4 The mediating role of savings rates

A lack of funds is one significant constraint for SMEs seeking digital transformation. Studies show that increases in the social savings rate can increase private sector investment and thus promote capital deepening [44]. As the savings rate rises, the scale of deposits that can be transformed into investment in society will increase accordingly, which is conducive to alleviating financial constraints, thereby promoting SMEs’ digital transformation. Then, how will population aging affect the savings rate? In theory, there are positive and negative aspects. On the one hand, the elderly population belongs to the “negative saving” group, and the increase in the proportion of the elderly population will reduce the social savings rate [45]. On the other hand, population aging is accompanied by longer lifespans and fewer children, and to prepare for retirement, people will save more when they are young, which may lead to a rise in the savings rate [46]. Additionally, the impact of population aging on savings rates will vary by the stage of aging and the policy environment. For example, in the early stages of aging, the proportion of the young population is much larger than that of the elderly population, which leads to a larger positive than negative effect of aging on the savings rate, thereby raising the savings rate. However, in the later stages of aging, the proportion of the elderly population significantly increases, which results in the negative effect outweighing the positive effect, thus decreasing the overall savings rate. Furthermore, establishing a mandatory pension system and providing tax incentives for individual contributions could also raise the savings rate. China is in the middle stage of aging where the proportion of the young population is still larger than that of the elderly population. In the meantime, China has established a basic social pension insurance system and is expanding coverage. Thus, in present China, it is expected that the positive effect of aging is greater than the negative effect and many studies support this view [42, 47]. Therefore, the following can be hypothesized.

Hypothesis 4: Population aging can affect SMEs by the pull effect of raising savings rates and thus positively impact the digital transformation of SMEs.

## 4. Research design

### 4.1 Variable selection

#### 4.1.1 Dependent variable

The dependent variable is the degree of digital transformation of the SMEs. There is no unified measurement standard for such an index. Since the frequency of digital-related words in annual reports disclosed by SMEs can reflect the importance of digital strategy for enterprises, we choose keyword frequency as the measure of this index. First, we construct a more comprehensive keyword dictionary. Referring to the existing studies of Wu et al. [48] and Zhao et al. [49], the keywords measuring enterprise digital transformation are divided into two categories: “bottom-level digital technology” and “digital technology application”, totaling 169 keywords. The bottom-level digital technology dimension is divided into four subdimensions: big data, cloud computing, artificial intelligence, and blockchain. The digital technology application dimension is also divided into four subdimensions: internet business model, industrial digitalization and intelligence, digital financial services, and modern information systems (see S1 Appendix). Second, Python is used to download the annual reports of NEEQ-listed enterprises and segment the text information. Third, we count the keyword frequency in annual report segments. Considering the differences in the text length of different annual reports, the sums of keyword frequencies are divided by the length of annual report segments to obtain the index. For ease of expression, this paper multiplies the index by 100. The larger the index is, the higher the degree of digital transformation of SMEs.

#### 4.1.2 Explanatory variable

The explanatory variable is the degree of population aging. The acceleration of population aging is reflected in two aspects: a rise in the proportion of the elderly population and an increase in the burden of dependency of the elderly population. Therefore, both the proportion of the population aged 65 and above in the total population and the proportion of the population aged 65 and above in the working-age population (15–64 years old) are used to measure it.

#### 4.1.3 Control variable

Given the existing studies, we select firm-level and regional-level control variables. The firm-level control variables include enterprise age (*Age*), enterprise size (*Size*), return on total assets (*Roa*), leverage ratio (*Lev*), fixed asset ratio (*Fix*), first largest shareholder holding ratio (*Shr*), revenue growth (*Growth*), operating cash flow ratio (*Cashflow*) and R&D investment ratio (*RD*). These variables may affect enterprise decision-making in the digital field, thus affecting enterprise digital transformation. The regional-level control variables include the urbanization level (*Urban*) and per capita GDP (*GDP*) of the city where the enterprise is registered. The definitions of the main variables are provided in Table 1.

**Table 1:** Definitions of primary variables.

Type	Symbol	Name	Definition
Dependent variable	Digital	Digital transformation degree	Digital transformation degree index*100
Explanatory variable	AR	Elderly population proportion	Number of people aged 65 and above/total population
	AFR	Elderly dependency ratio	Number of people aged 65 and above/total population aged 15–64
Control variable	Age	Enterprise age	Ln(observation year-establishment year+1)
	Size	Enterprise scale	Ln(total assets)
	Roa	Return on total assets	Net profit/total assets
	Lev	Leverage ratio	Total liabilities/total assets
	Fix	Fixed asset ratio	Total Fixed assets/total assets
	Shr	First largest shareholder holding ratio	First largest shareholder’s shareholding amount/total share capital
	Growth	Revenue growth	Year-on-year growth in operating income
	Cashflow	Operating cash flow ratio	Net cash flow from operations/total assets
	RD	R&D investment ratio	Total R&D expenditure/total assets
	Urban	Urbanization level	Resident population/total population
	GDP	Economic growth	Ln(per capita GDP)

### 4.2 Data sources

**Fig 2:**
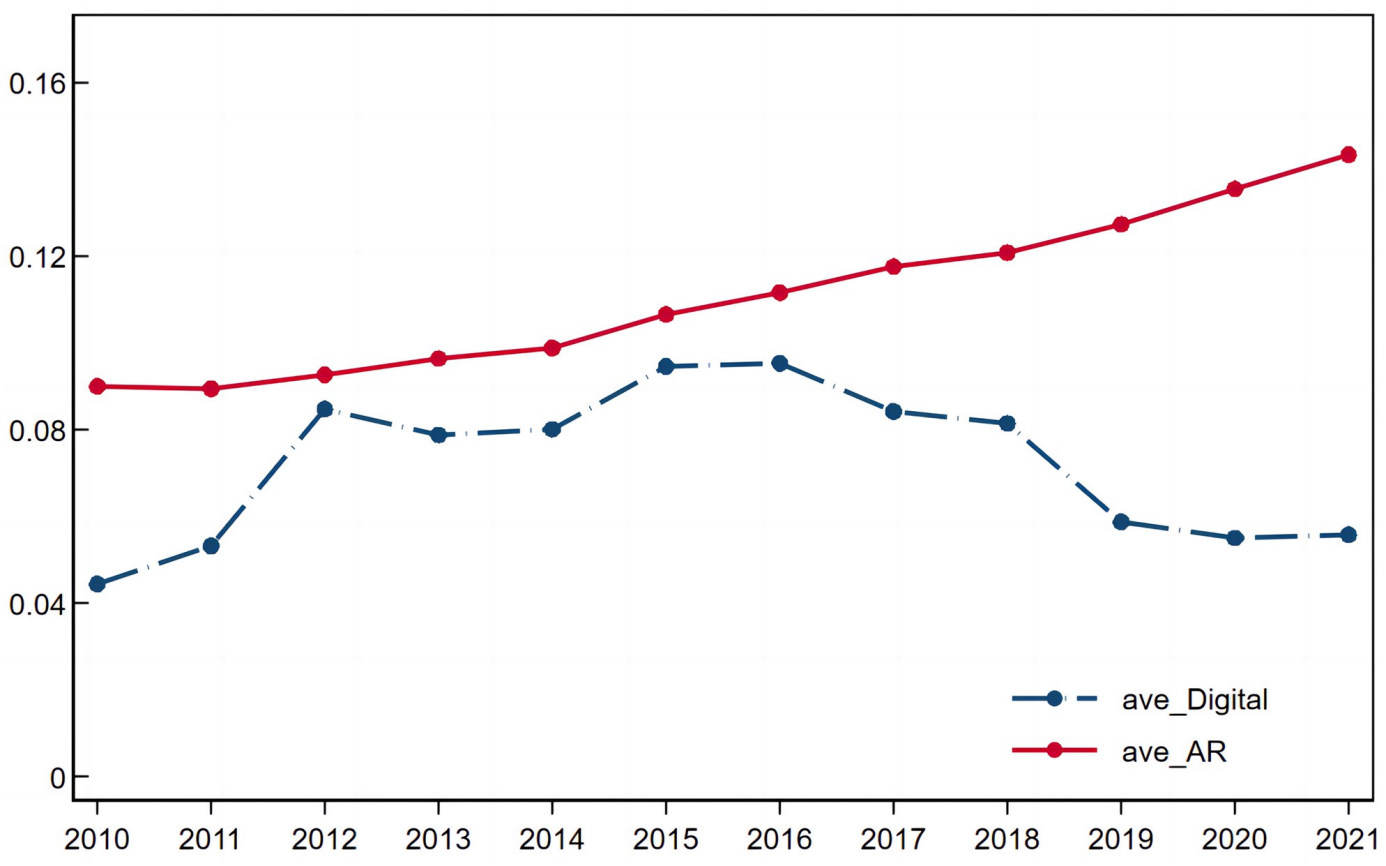
Trends of population aging and SME digital transformation.

This paper selects NEEQ-listed enterprises from 2010 to 2021 in China as samples and obtains unbalanced panel data containing 7,700 enterprises after excluding financial enterprises, ST enterprises and enterprises with severe missing data. The main sources of the data are as follows: (1) the enterprise micro data are from the Wind database and the CSMAR database, (2) the enterprise digital transformation indices are derived from textual analytics, and (3) the macro data of 31 provinces in China are from the China Statistical Yearbook, China Population Statistics Yearbook, China Education Statistics Yearbook, and Provincial Statistical Yearbook. To alleviate the influence of extreme values, all of the continuous variables are winsorized at the 1% and 99% levels. The descriptive statistics of the main variables are shown in Table 2, and the trends of population aging and SME digital transformation are presented in Fig 2.

**Table 2:** Descriptive statistics of primary variables.

Variable	Mean	SD	Min	P50	Max	Number
Digital	0.07	0.12	0.00	0.03	1.61	40655
AR	0.11	0.03	0.07	0.11	0.17	85319
AFR	0.15	0.04	0.09	0.15	0.25	85319
Age	2.17	0.70	0.00	2.30	4.03	80808
Size	18.23	1.27	14.91	18.28	21.31	60942
Roa	0.03	0.15	-0.65	0.04	0.40	61016
Lev	0.45	0.24	0.03	0.43	1.23	60975
Fix	0.16	0.16	0.00	0.11	0.68	60437
Shr	0.51	0.20	0.15	0.50	0.98	47026
Growth	0.27	0.79	-0.82	0.11	5.27	47505
Cashflow	0.03	0.16	-0.57	0.03	0.47	60936
RD	0.06	0.06	0.00	0.04	0.39	48953
Urban	0.67	0.14	0.40	0.66	0.90	84937
GDP	11.14	0.47	9.96	11.16	12.06	85319

### 4.3 Model construction

To test the effect of population aging on SME digital transformation, the following model is established for empirical research:

$${\;Digital}_{r,i,t}=\varphi_{0}+\varphi_{1}{AR}_{r,t}+\varphi_{2}X_{i,t}+\varphi_{3}Z_{r,t}+\vartheta_{i}+\delta_{t}+\varepsilon_{i,t}$$

where *Digital*_*r*,*i*,*t*_ is the digital transformation index of firm i in province r in year t; *AR*_*r*,*t*_ is the degree of population aging in the province where the firm is located; *X*_*i*,*t*_ represents firm-level control variables; *Z*_*r*,*t*_ represents regional-level control variables; *υ*_*i*_ and *δ*_*t*_ represent firm fixed effects and year fixed effects, respectively; and *ε*_*i*,*t*_ represents the random error term.

## 5. Results and discussion

### 5.1 Baseline regression analysis

Table 3 reports the results of the baseline regression. The explanatory variable in columns (1) and (3) is the proportion of the elderly population, while the explanatory variable in columns (2) and (4) is the elderly dependency ratio. The results show that when no control variables are included (columns (1) and (2)), the coefficients of both the elderly population proportion and the elderly dependency ratio are significantly positive at the 1% level. After adding control variables in the regression (columns (3) and (4)), the regression coefficient of the proportion of the elderly population is 0.1568 and passes the 1% significance test, while that of the elderly dependency ratio is 0.0970 and passes the 1% significance test. The above results indicate that the current process of population aging in China has a positive effect on SME digital transformation, thus confirming Hypothesis 1.

**Table 3:** Baseline regression results.

	(1)	(2)	(3)	(4)
Variable	Digital	Digital	Digital	Digital
AR	0.2113***		0.1568***	
	(3.2527)		(4.7002)	
AFR		0.1507***		0.0970***
		(3.0414)		(4.2597)
Age			-0.0267	-0.0267
			(-1.7260)	(-1.7259)
Size			0.0143**	0.0143**
			(2.8461)	(2.8500)
Roa			-0.0059	-0.0059
			(-0.7658)	(-0.7671)
Lev			-0.0316***	-0.0315***
			(-3.2417)	(-3.2399)
Fix			0.0106	0.0106
			(1.3748)	(1.3706)
Shr			-0.0011	-0.0011
			(-0.1359)	(-0.1418)
Growth			-0.0001	-0.0001
			(-0.0544)	(-0.0551)
Cashflow			-0.0029	-0.0029
			(-1.0535)	(-1.0561)
RD			0.0672***	0.0671***
			(4.9003)	(4.8952)
Urban			0.1589***	0.1507***
			(3.2356)	(3.1703)
GDP			-0.0192	-0.0189
			(-1.5283)	(-1.5158)
Constant	0.0450***	0.0452***	-0.0243	-0.0193
	(5.5980)	(5.3265)	(-0.3759)	(-0.3022)
Firm	controlled	controlled	controlled	controlled
Year	controlled	controlled	controlled	controlled
Observations	40,425	40,425	28,498	28,498
AdjR-squared	0.8127	0.8128	0.8436	0.8436

Note:

*, **, and *** are the significance levels at 10%, 5%, and 1%, respectively; t statistics in parentheses. Same for below.

### 5.2 Robustness test

#### 5.2.1 Disaggregated explained variables

For the robustness of the results, this paper disaggregates the total index of SMEs’ digital transformation degree (*Digital*) into two subindices: bottom-level digital technology (*Digital_tech*) and digital technology application (*Digital_apply*) for retesting. The results are reported in Table 4. Both subindices pass the 10% significance test, indicating that after changing the measurement method of digital transformation, the conclusion that population aging positively impacts the digital transformation of SMEs still holds, thus verifying the robustness of the results.

**Table 4:** Robustness test of disaggregated explained variables.

Variable	Digital_tech	Digital_tech	Digital_apply	Digital_apply
AR	0.0239*		0.0170**	
	(2.1043)		(2.4711)	
AFR		0.0141*		0.0115*
		(1.9672)		(1.9846)
Age	-0.0008	-0.0008	0.0015	0.0015
	(-0.9492)	(-0.9435)	(0.6239)	(0.6244)
Size	0.0021**	0.0021**	0.0010**	0.0010**
	(2.1852)	(2.1869)	(2.8983)	(2.8757)
Roa	-0.0001	-0.0001	0.0022**	0.0022**
	(-0.1237)	(-0.1254)	(2.3597)	(2.3572)
Lev	-0.0015**	-0.0015**	-0.0079**	-0.0079**
	(-2.3009)	(-2.3017)	(-2.1266)	(-2.1278)
Fix	0.0018	0.0018	0.0031***	0.0031***
	(1.1861)	(1.1845)	(4.7211)	(4.7041)
Shr	0.0008*	0.0008*	0.0004	0.0004
	(2.0996)	(2.0922)	(0.1645)	(0.1608)
Growth	-0.0003	-0.0003	-0.0006	-0.0006
	(-1.6452)	(-1.6459)	(-1.6827)	(-1.6819)
Cashflow	-0.0003	-0.0003	0.0003	0.0003
	(-1.5776)	(-1.5880)	(0.7149)	(0.7136)
RD	0.0088**	0.0088**	0.0062*	0.0062*
	(2.8102)	(2.8069)	(1.8230)	(1.8082)
Urban	0.0172**	0.0161**	0.0331***	0.0321***
	(2.5427)	(2.5549)	(3.8969)	(3.8936)
GDP	-0.0013	-0.0012	-0.0068	-0.0067
	(-0.5832)	(-0.5728)	(-1.3795)	(-1.3830)
Constant	-0.0330***	-0.0321***	0.0469	0.0472
	(-6.4164)	(-6.3376)	(0.9025)	(0.9160)
Firm	controlled	controlled	controlled	controlled
Year	controlled	controlled	controlled	controlled
Observations	28,498	28,498	28,498	28,498
AdjR-squared	0.7925	0.7925	0.8379	0.8379

#### 5.2.2 Alternative explained variable

Referring to Li et al. [50], we use the Chinese pre-trained BERT model to construct the word vector for each initial digital transformation keyword. Subsequently, we extract words from the annual report vocabulary that have 80% similarity to the digital transformation word vectors and filter 42 additional keywords representing the digital transformation of SMEs. Finally, we use the expanded 118 keywords to construct *Digital_2* and then replace *Digital* with *Digital_2* for retesting. The results are shown in Table 5, which are consistent with the baseline results, thus confirming the robustness of the results.

**Table 5:** Robustness test of alternative explained variables.

	(1)	(2)	(3)	(4)
Variable	Digital_2	Digital_2	Digital_2	Digital_2
AR	0.2721***		0.2316***	
	(3.0662)		(3.4064)	
AFR		0.2201***		0.1456***
		(2.9666)		(3.6060)
Age			-0.0553	-0.0553
			(-1.3479)	(-1.3476)
Size			0.0166*	0.0166*
			(1.9020)	(1.9044)
Roa			0.0147	0.0147
			(1.3840)	(1.3810)
Lev			-0.0438**	-0.0438**
			(-2.3945)	(-2.3936)
Fix			0.0248***	0.0247***
			(3.7090)	(3.6965)
Shr			0.0392**	0.0391**
			(2.8908)	(2.8778)
Growth			0.0027**	0.0027**
			(2.8644)	(2.8634)
Cashflow			-0.0057	-0.0057
			(-1.4155)	(-1.4159)
RD			0.1513***	0.1512***
			(6.5597)	(6.5569)
Urban			0.4277**	0.4153**
			(2.2626)	(2.2082)
GDP			-0.0184	-0.0179
			(-1.0135)	(-0.9952)
Constant	0.3124***	0.3083***	0.0304	0.0373
	(28.4824)	(24.2447)	(0.2905)	(0.3668)
Firm	controlled	controlled	controlled	controlled
Year	controlled	controlled	controlled	controlled
Observations	40,415	40,415	28,491	28,491
AdjR-squared	0.8352	0.8353	0.8503	0.8504

#### 5.2.3 Sample selection transformation

To eliminate possible estimation bias caused by sample selection bias, we retest by replacing samples. Most studies use data at the provincial level, with insufficient analysis of data at the city level. Hence, this paper selects population aging data at the city level in Anhui, Gansu, Henan, and Jiangxi provinces, which have the most complete data among all of the provinces. The results are listed in Table 6. The conclusion that population aging positively impacts SME digital transformation is significant, based on both provincial and city data, thus checking the robustness of the results.

**Table 6:** Robustness test of transformed sample ranges.

Variable	Digital	Digital
AR	0.3199*	
	(2.0186)	
AFR		0.0816*
		(1.8705)
Age	0.0147	0.0256
	(0.7954)	(0.7806)
Size	0.0061**	0.0056
	(2.6301)	(0.8861)
Roa	0.0099	0.0100
	(0.9280)	(1.1266)
Lev	-0.0062	-0.0099**
	(-0.8722)	(-2.3202)
Fix	0.0103	0.0086
	(0.8867)	(1.3448)
Shr	-0.0055	-0.0297***
	(-1.1383)	(-3.2009)
Growth	-0.0035	0.0049
	(-0.4472)	(1.5002)
Cashflow	-0.0181	-0.0223**
	(-1.2800)	(-2.4104)
RD	0.0643*	0.0524
	(1.8506)	(1.3664)
Urban	0.3349	0.9366***
	(0.7838)	(4.3422)
GDP	0.0415	0.0441**
	(1.1332)	(2.2146)
Constant	-0.7561**	-1.1068***
	(-3.0511)	(-3.3094)
Firm	controlled	controlled
Year	controlled	controlled
Observations	2,522	1,428
AdjR-squared	0.8709	0.8077

### 5.3 Endogeneity analysis

#### 5.3.1 Lagging explanatory variables

To eliminate the endogeneity problem due to reverse causality, this paper lags the explanatory variables for one, two, and three periods. The results are provided in Table 7. The coefficients of the explanatory variables lagged by one period and two periods are both significantly positive and pass the 1% significance test, while the coefficients of the explanatory variables lagged by three periods are also both significantly positive and pass the 10% significance test. This reveals that the impact of population aging on SME digital transformation has a lag effect and tests the robustness of the results.

**Table 7:** Endogenous analysis of lagged explanatory variables.

Variable	Digital	Digital	Digital	Digital	Digital	Digital
L.AR	0.1947***					
	(3.4433)					
L.AFR		0.1184***				
		(3.8329)				
L2.AR			0.2899***			
			(3.9642)			
L2.AFR				0.1777***		
				(4.1004)		
L3.AR					0.1120*	
					(1.8469)	
L3.AFR						0.0727*
						(1.9084)
Age	-0.0137	-0.0137	-0.0060	-0.0060	0.0106**	0.0106**
	(-1.6124)	(-1.6124)	(-1.0355)	(-1.0355)	(2.1382)	(2.1462)
Size	0.0140***	0.0140***	0.0084**	0.0084***	0.0055**	0.0055**
	(3.2353)	(3.2414)	(2.9069)	(2.9210)	(2.7050)	(2.7110)
Roa	0.0132	0.0132	0.0270***	0.0270***	0.0155***	0.0155***
	(1.1889)	(1.1852)	(4.0768)	(4.0593)	(4.3447)	(4.3444)
Lev	-0.0244***	-0.0244***	-0.0143**	-0.0143**	0.0026	0.0026
	(-4.3335)	(-4.3331)	(-2.9035)	(-2.9162)	(1.0699)	(1.0652)
Fix	0.0134	0.0134	0.0129*	0.0128*	-0.0046	-0.0046
	(1.4639)	(1.4582)	(1.8579)	(1.8535)	(-0.5241)	(-0.5264)
Shr	0.0080	0.0080	0.0157	0.0157	0.0033	0.0034
	(0.6639)	(0.6607)	(0.7676)	(0.7675)	(0.3279)	(0.3289)
Growth	0.0008*	0.0008*	0.0014	0.0015	0.0012	0.0012
	(1.8811)	(1.8784)	(1.5951)	(1.6019)	(1.5927)	(1.5940)
Cashflow	0.0004	0.0004	-0.0007	-0.0007	-0.0040	-0.0040
	(0.2109)	(0.2038)	(-0.2793)	(-0.2816)	(-1.6104)	(-1.6137)
RD	0.0658**	0.0658**	0.0327*	0.0327*	0.0186***	0.0185***
	(2.5920)	(2.5876)	(1.9258)	(1.9230)	(3.3602)	(3.3517)
Urban	0.1906**	0.1804**	0.2816***	0.2666***	0.1176***	0.1116***
	(2.7120)	(2.5571)	(3.0498)	(2.9353)	(5.3754)	(5.0923)
GDP	-0.0269***	-0.0265***	-0.0171***	-0.0166***	-0.0011	-0.0008
	(-2.9829)	(-2.9481)	(-7.4460)	(-7.2160)	(-0.1464)	(-0.0961)
Constant	-0.0024	0.0045	-0.1089	-0.0992	-0.1401	-0.1385
	(-0.0727)	(0.1384)	(-0.9246)	(-0.8310)	(-1.0737)	(-1.0466)
Firm	controlled	controlled	controlled	controlled	controlled	controlled
Year	controlled	controlled	controlled	controlled	controlled	controlled
Observations	28,733	28,733	23,391	23,391	17,786	17,786
AdjR-squared	0.8400	0.8400	0.8613	0.8613	0.8922	0.8922

#### 5.3.2 Instrumental variable approach

In addition, this paper uses the instrumental variable method to alleviate the endogeneity problem. Referring to Cai et al. [51], we select the birth rate in 1980 of the province where the enterprise is located as the instrumental variable for the degree of population aging from 2010 to 2021. This instrumental variable was chosen because the historical birth rate has good predictive ability for the degree of population aging, and it is difficult for the birth rate 30 years ago to directly influence current decisions on enterprise digital transformation. The results are presented in Table 8. There is still a significant positive relationship between population aging and SMEs’ digital transformation after retesting with the instrumental variable. We also report the results of a weak instrumental variable test at the end of Table 8, and the statistical values are much larger than 100, which supports the rationality of the instrumental variable selection.

**Table 8:** Endogenous analysis of instrumental variables.

Variable	Digital	Digital
AR	0.0851*	
	(1.8032)	
AFR		0.0626*
		(1.8026)
Age	-0.0120***	-0.0121***
	(-7.5903)	(-7.6080)
Size	0.0073***	0.0073***
	(9.3874)	(9.3686)
Roa	-0.0182***	-0.0181***
	(-2.7652)	(-2.7394)
Lev	-0.0294***	-0.0295***
	(-8.6884)	(-8.7003)
Fix	-0.1866***	-0.1866***
	(-41.1079)	(-41.0902)
Shr	-0.0211***	-0.0211***
	(-6.2942)	(-6.3034)
Growth	0.0077***	0.0077***
	(5.6586)	(5.6708)
Cashflow	-0.0410***	-0.0412***
	(-6.6791)	(-6.7004)
RD	0.4195***	0.4193***
	(22.6880)	(22.6798)
Urban	0.1096***	0.1147***
	(8.2938)	(7.9096)
GDP	-0.0372***	-0.0379***
	(-9.6369)	(-9.3231)
Constant	0.3376***	0.3423***
	(9.5173)	(9.3711)
Firm	controlled	controlled
Year	controlled	controlled
KP-F	13230.18	9259.15
Observations	28,594	28,594
AdjR-squared	0.1159	0.1154

### 5.4 Heterogeneity analysis

#### 5.4.1 Regional heterogeneity

There are visible differences in economic development between the eastern and central-western regions in China. Therefore, according to the locations of enterprises, this paper divides the samples into eastern and central-western regions. The regression results in Table 9 show that the digital transformation coefficient is positively significant at the 5% level for enterprises in eastern regions but is not significant for those in central-western regions. One possible explanation is that compared with central-western regions, eastern regions have developed economies, advanced technologies, abundant financial resources, and higher population quality. Therefore, the labor substitution effect of capital and technology, the human capital accumulation effect, and the raising savings rate effect caused by population aging are greater. Thus, the promotion effects on SME digital transformation are also greater in the eastern regions of China.

**Table 9:** Heterogeneous analysis of regions.

	Eastern Regions		Central-western Regions	
Variable	Digital	Digital	Digital	Digital
AR	0.1202**		0.0270	
	(2.5607)		(0.3175)	
AFR		0.0786**		0.0104
		(2.7191)		(0.1895)
Age	-0.0352**	-0.0352**	-0.0069	-0.0068
	(-2.5857)	(-2.5853)	(-0.3720)	(-0.3698)
Size	0.0141***	0.0140***	0.0151**	0.0152**
	(3.0620)	(3.0656)	(2.4645)	(2.4644)
Roa	-0.0097	-0.0098	0.0049	0.0048
	(-1.1234)	(-1.1262)	(0.4700)	(0.4717)
Lev	-0.0330***	-0.0330***	-0.0267*	-0.0268*
	(-4.0903)	(-4.0841)	(-1.8081)	(-1.8118)
Fix	0.0153	0.0153	0.0027	0.0026
	(1.1509)	(1.1503)	(0.5008)	(0.4954)
Shr	0.0001	0.0001	-0.0023	-0.0022
	(0.0124)	(0.0054)	(-0.4601)	(-0.4280)
Growth	0.0001	0.0001	-0.0009	-0.0009
	(0.1487)	(0.1481)	(-0.4292)	(-0.4293)
Cashflow	0.0048	0.0049	-0.0259**	-0.0259**
	(0.8285)	(0.8301)	(-2.2390)	(-2.2456)
RD	0.0514***	0.0513***	0.1465**	0.1468**
	(4.8803)	(4.8797)	(2.6887)	(2.6944)
Urban	0.1255	0.1186	-0.1718	-0.1697
	(1.5290)	(1.4789)	(-0.9042)	(-0.8994)
GDP	-0.0300*	-0.0294*	-0.0043	-0.0044
	(-1.9953)	(-1.9780)	(-0.7138)	(-0.7354)
Constant	0.1553*	0.1557*	-0.0515	-0.0473
	(1.8821)	(1.9250)	(-0.4816)	(-0.4514)
Firm	controlled	controlled	controlled	controlled
Year	controlled	controlled	controlled	controlled
Observations	20,215	20,215	8,283	8,283
AdjR-squared	0.8372	0.8372	0.8616	0.8616

#### 5.4.2 Corporate heterogeneity

To explore the impact of population aging on SME digital transformation under different types of ownership, this paper divides the samples into state-owned enterprises and private enterprises. The estimation results in Table 10 show that the impact is significantly positive for enterprises with state-owned ownership but is not significant for private enterprises. This suggests that population aging has a stronger driving force for private enterprise digital transformation. The reason is that state-owned enterprises have advantages in acquiring resources such as capital, talent, and technology, as well as in market competitiveness. Consequently, the marginal impacts of higher labor costs, increased human capital, and higher savings rates caused by population aging are relatively small. In contrast, private enterprises that have poor financial environments and weak abilities to access resources such as talent and technology are more affected by the above effects, thus having a larger incentive to digitally transform.

**Table 10:** Heterogeneous analysis of firm nature.

	State-owned Enterprises		Private Enterprises	
Variable	Digital	Digital	Digital	Digital
AR	0.2185		0.1467***	
	(1.2037)		(5.5491)	
AFR		0.1378		0.0913***
		(1.2315)		(5.1417)
Age	0.0052	0.0052	-0.0269	-0.0269
	(0.2234)	(0.2251)	(-1.6775)	(-1.6776)
Size	0.0116	0.0115	0.0144***	0.0144***
	(1.6403)	(1.6277)	(3.0083)	(3.0128)
Roa	-0.0220	-0.0220	-0.0047	-0.0047
	(-0.6132)	(-0.6124)	(-0.6507)	(-0.6521)
Lev	-0.0375***	-0.0375***	-0.0308***	-0.0308***
	(-3.0758)	(-3.0753)	(-3.3419)	(-3.3396)
Fix	0.0025	0.0026	0.0114	0.0113
	(0.1585)	(0.1684)	(1.3269)	(1.3231)
Shr	0.0286	0.0284	-0.0037	-0.0037
	(1.0063)	(1.0011)	(-0.4928)	(-0.4996)
Growth	0.0027	0.0027	-0.0002	-0.0002
	(1.1805)	(1.1814)	(-0.2051)	(-0.2061)
Cashflow	0.0283	0.0282	-0.0040	-0.0040
	(1.2196)	(1.2161)	(-1.7017)	(-1.7025)
RD	-0.0687	-0.0696	0.0700***	0.0699***
	(-0.9309)	(-0.9445)	(5.5516)	(5.5446)
Urban	0.0959	0.0848	0.1502***	0.1424***
	(0.5923)	(0.5276)	(3.3677)	(3.2735)
GDP	0.0178	0.0182	-0.0215	-0.0212
	(0.8072)	(0.8214)	(-1.5248)	(-1.5114)
Constant	-0.4609	-0.4522	0.0092	0.0137
	(-1.3498)	(-1.3325)	(0.1033)	(0.1549)
Firm	controlled	controlled	controlled	controlled
Year	controlled	controlled	controlled	controlled
Observations	1,371	1,371	27,106	27,106
AdjR-squared	0.9254	0.9254	0.8406	0.8406

## 6. Influence mechanism analysis

### 6.1 Test of the mediating role of labor costs

To verify the mediating effect of labor costs, following Wang et al. [52], the relative wage of the province’s average wage level compared to that of the country is used as a measure of relative labor costs. The results are listed in Table 11. Columns (1) and (3) show that population aging has a significant positive impact on the relative average wage level, which means that population aging increases labor costs. In columns (2) and (4), after adding the mediating variable of the relative average wage level, the impact of population aging on enterprise digital transformation is still positive, which means that population aging positively impacts SME digital transformation by increasing labor costs, thus proving Hypothesis 2. This is because population aging leads to higher labor costs. Under the burden of higher labor costs, SMEs are pushed to replace labor with capital and technology, leading to more digital transformation.

**Table 11:** Mechanism analysis of the effect of population aging on SME digital transformation by increasing labor costs.

	(1)	(2)	(3)	(4)
Variable	Wage	Digital	Wage	Digital
Wage		0.0059***		0.0059***
		(4.0895)		(4.1659)
AR	16.0081***	0.0579*		
	(8.9202)	(1.9966)		
AFR			9.6136***	0.0385**
			(8.4269)	(2.3168)
Age	0.0001	-0.0267*	0.0015	-0.0267*
	(0.0015)	(-1.7700)	(0.0342)	(-1.7690)
Size	0.0227***	0.0142**	0.0229***	0.0142**
	(3.4618)	(2.8345)	(3.5837)	(2.8371)
Roa	-0.0022	-0.0060	-0.0037	-0.0060
	(-0.1516)	(-0.7812)	(-0.2583)	(-0.7819)
Lev	-0.0320	-0.0316***	-0.0322	-0.0316***
	(-1.3031)	(-3.2030)	(-1.3091)	(-3.2034)
Fix	-0.0080	0.0106	-0.0104	0.0106
	(-0.2060)	(1.4117)	(-0.2606)	(1.4087)
Shr	-0.0748***	-0.0007	-0.0767***	-0.0007
	(-3.2565)	(-0.0845)	(-3.3001)	(-0.0886)
Growth	-0.0005	-0.0000	-0.0004	-0.0000
	(-0.2107)	(-0.0418)	(-0.1773)	(-0.0424)
Cashflow	-0.0362**	-0.0026	-0.0368**	-0.0026
	(-2.7636)	(-0.9541)	(-2.7616)	(-0.9549)
RD	0.1306	0.0663***	0.1313	0.0663***
	(1.2220)	(4.6935)	(1.2964)	(4.6977)
Urban	37.4866***	-0.0643**	36.6619***	-0.0657**
	(17.4854)	(-2.3574)	(17.4809)	(-2.5864)
GDP	-1.8749***	-0.0080	-1.8489***	-0.0080
	(-11.1047)	(-0.8506)	(-11.0415)	(-0.8419)
Constant	-8.1177***	0.0237	-7.5158***	0.0247
	(-18.0900)	(0.3303)	(-15.8599)	(0.3531)
Firm	controlled	controlled	controlled	controlled
Year	controlled	controlled	controlled	controlled
Observations	32,528	28,498	32,528	28,498
AdjR-squared	0.9907	0.8438	0.9907	0.8438

### 6.2 Test of the mediating role of human capital accumulation

To verify the mediating effect of human capital, following Guo et al. [53], the proportion of students in higher education to the total population is used as a measure of human capital. The results are provided in Table 12. Columns (1) and (3) show that population aging has a significant positive impact on human capital, which means that population aging can facilitate human capital accumulation. Columns (2) and (4) regress both human capital level and aging degree into the model, and the coefficient of enterprise digital transformation is still significantly positive, indicating that population aging positively impacts enterprise digital transformation by facilitating human capital accumulation, thus proving Hypothesis 3. The reason is that population aging means longer life expectancy and higher return on educational investment, thus promoting human capital accumulation. As the quality of the workforce improves, enterprise demand for highly skilled laborers can be better met, which makes SMEs obtain the pull from increasing human capital for digital transformation.

**Table 12:** Mechanism analysis of the effect of population aging on SME digital transformation by facilitating human capital accumulation.

	(1)	(2)	(3)	(4)
Variable	Edu	Digital	Edu	Digital
Edu		0.0983**		0.0975**
		(2.6350)		(2.6321)
AR	0.4135***	0.1062***		
	(5.7413)	(3.7931)		
AFR			0.2752***	0.0644***
			(6.4966)	(3.5465)
Age	0.0039	-0.0295*	0.0039	-0.0295*
	(0.9989)	(-1.8871)	(0.9942)	(-1.8871)
Size	-0.0005	0.0137***	-0.0005	0.0137***
	(-0.2617)	(3.1462)	(-0.2723)	(3.1486)
Roa	-0.0001	-0.0078	-0.0002	-0.0078
	(-0.0370)	(-0.9834)	(-0.0636)	(-0.9840)
Lev	-0.0033**	-0.0327***	-0.0032**	-0.0327***
	(-2.2480)	(-3.1678)	(-2.2608)	(-3.1681)
Fix	-0.0036	0.0109	-0.0036	0.0109
	(-1.2233)	(1.3430)	(-1.2151)	(1.3407)
Shr	0.0004	-0.0020	0.0002	-0.0020
	(0.2050)	(-0.2740)	(0.1253)	(-0.2785)
Growth	0.0005**	0.0000	0.0005**	0.0000
	(2.6789)	(0.0320)	(2.7076)	(0.0322)
Cashflow	-0.0027	-0.0017	-0.0027	-0.0017
	(-1.0874)	(-0.6087)	(-1.0863)	(-0.6116)
RD	-0.0054	0.0658***	-0.0056	0.0658***
	(-0.5825)	(5.1999)	(-0.6015)	(5.1950)
Urban	0.5022***	0.0962**	0.4777***	0.0911**
	(9.5403)	(2.4731)	(9.7433)	(2.4137)
GDP	-0.0004	-0.0209	0.0008	-0.0207
	(-0.1299)	(-1.5356)	(0.2416)	(-1.5282)
Constant	-0.1474***	0.0396	-0.1393***	0.0434
	(-5.7936)	(0.4226)	(-4.8967)	(0.4662)
Firm	controlled	controlled	controlled	controlled
Year	controlled	controlled	controlled	controlled
Observations	31,351	27,480	31,351	27,480
AdjR-squared	0.9925	0.8426	0.9925	0.8426

### 6.3 Test of the mediating role of savings rates

To verify the mediating effect of savings rates, following Xu et al. [54], the proportion of total savings to regional GDP is used as a measure of the savings rate. In Table 13, columns (1) and (3) show the impact of population aging on the savings rate. At the 5% level, the coefficient is significantly positive, indicating that population aging can raise savings rates. Columns (2) and (4) display the results of the regression after adding the savings rate. At the 10% level, the regression coefficient of population aging and enterprise digital transformation is significant, which means that population aging positively impacts enterprise digital transformation by raising savings rates, thus verifying Hypothesis 4. This result can be explained by the following reasons. First, China is in the middle stage of aging, and the proportion of the young population is still much larger than that of the elderly population. Second, China has an expanding basic social pension insurance system. Therefore, population aging has a positive effect on savings rates in China at this stage. As the savings rate rises, creditable funds in the financial market increase, which makes SMEs obtain the pull from alleviating financial constraints for digital transformation.

**Table 13:** Mechanism analysis of the effect of population aging on SME digital transformation by raising the savings rate.

	(1)	(2)	(3)	(4)
Variable	Save	Digital	Save	Digital
Save		0.0257**		0.0251**
		(2.0720)		(2.4825)
AR	0.7746**	0.1946*		
	(2.6764)	(1.9146)		
AFR			0.5581**	0.1237*
			(2.6682)	(1.9648)
Age	-0.0057	-0.0566***	-0.0062	-0.0566***
	(-0.8186)	(-3.6400)	(-0.8809)	(-3.7601)
Size	-0.0000	0.0173***	-0.0001	0.0173***
	(-0.0128)	(4.9813)	(-0.0661)	(4.4078)
Roa	-0.0058	-0.0066	-0.0059	-0.0066
	(-1.5627)	(-0.6871)	(-1.6037)	(-0.4964)
Lev	-0.0060*	-0.0375***	-0.0059*	-0.0375***
	(-2.0269)	(-5.2126)	(-1.9505)	(-4.8754)
Fix	-0.0068	0.0481***	-0.0067	0.0481*
	(-0.6960)	(3.6100)	(-0.6867)	(1.9958)
Shr	0.0002	-0.0020	0.0003	-0.0020
	(0.0651)	(-0.1183)	(0.0901)	(-0.3150)
Growth	0.0003	0.0010	0.0002	0.0010
	(0.3638)	(1.0146)	(0.3551)	(1.2737)
Cashflow	0.0011	0.0119	0.0013	0.0119*
	(0.4812)	(1.6275)	(0.5903)	(2.0553)
RD	-0.0017	0.0428*	-0.0022	0.0428**
	(-0.2047)	(1.8262)	(-0.2664)	(2.3967)
Urban	0.6472***	0.2338**	0.6180***	0.2271***
	(13.4979)	(2.0593)	(12.1490)	(3.9602)
GDP	-0.5215***	-0.0125	-0.5215***	-0.0129*
	(-84.0587)	(-1.1673)	(-84.2294)	(-1.7578)
Constant	6.4409***	-0.1729	6.4736***	-0.1586
	(77.9307)	(-0.9110)	(80.4631)	(-1.0270)
Firm	controlled	controlled	controlled	controlled
Year	controlled	controlled	controlled	controlled
Observations	8,518	7,165	8,518	7,165
AdjR-squared	0.9170	0.8352	0.9173	0.8352

## 7. Further analysis

### 7.1 Threshold effect of aging degree

To study the impact of differing degrees of population aging on SME digital transformation, this paper uses the threshold effect model. As reported in Table 14, when the elderly population proportion is used as a threshold variable, there is a double threshold at the 5% significance level, and the threshold values are 12.5% and 15.01%. The test results in Table 15 show that when the proportion of the elderly population is lower than 12.5%, the coefficient of population aging on enterprise digital transformation is 0.5401. When the proportion of the elderly population is between 12.5% and 15.01%, the coefficient is 0.4105. When the proportion of the elderly population is higher than 15.01%, the coefficient is 0.2890. The above results reveal that although population aging has a positive impact on enterprise digital transformation, the marginal positive impact decreases as population aging progresses. One possible reason is that in the early stages of population aging, higher labor costs can be easily offset by technological progress, and the extension of life expectancy increases the savings rate, which results in a greater positive impact on enterprise digital transformation. However, in the later stages of population aging, severe labor shortages make the substitution effect of capital and technology negligible, and the upward trend of the social savings rate is slowed by increasing social pension payouts, which weakens the positive impact on digital transformation.

**Table 14:** Threshold regression test of population aging.

Threshold variable	Threshold type	F-value	P-value	Crit-value			Threshold value
				1%	5%	10%	
AR	Single Threshold	58.48	0.000	9.573	10.187	14.246	
	Double Threshold	71.50	0.027	19.984	58.685	80.235	0.1250, 0.1501

Note: Threshold values are the results obtained by repeatedly sampling 300 times using the bootstrap sampling method. Same for below.

**Table 15:** Double threshold regression results of population aging.

Variable	Digital
AR(AR<12.50%)	0.5401***
	(6.6756)
AR(12.50%≤AR<15.01%)	0.4105***
	(5.5512)
AR(AR≥15.01%)	0.2890***
	(4.1288)
Age	-0.0661***
	(-14.5255)
Size	0.0152***
	(10.1938)
Roa	-0.0047
	(-1.0788)
Lev	-0.0198***
	(-7.0987)
Fix	0.0208***
	(2.9088)
Shr	-0.0106
	(-1.1624)
Growth	-0.0001
	(-0.8625)
Cashflow	-0.0001
	(-0.0169)
RD	0.0402***
	(3.9921)
Urban	-0.0607*
	(-1.6519)
GDP	0.0001
	(0.2001)
Constant	-0.0443
	(-1.3452)
Observations	22,406
AdjR-squared	0.0552

### 7.2 Threshold effect of marketization level

To study the impact of population aging on SME digital transformation under different marketization levels, this paper selects the marketization process index in China’s Provincial Marketization Index Report (2021) [55] as a measure of the level of marketization. Since this report only includes marketization indices from 2010 to 2019, we extend the data to the whole sample period according to the annual average growth rate of the marketization index.

As presented in Table 16, when the marketization level is used as a threshold variable, two threshold values are obtained at 9.01 and 10.15. The test results in Table 17 show that when the marketization degree is at a low level (*Market*<9.01), the impact coefficient of population aging on enterprise digital transformation is 0.1401. When the marketization degree is at a medium level (9.01≤*Market*<10.15), the impact coefficient of population aging on enterprise digital transformation increases to 0.1924. When the marketization degree is at a high level (*Market*≥10.15), the impact coefficient of population aging on enterprise digital transformation increases to 0.2379. The above results suggest that with increasing marketization levels, the impact of population aging on enterprise digital transformation gradually increases. One possible reason is that enterprises become more sensitive to production factor supply and price changes as the marketization level increases. Therefore, changes in the supply and relative prices of production factors such as labor, human capital, and finance caused by population aging will have a greater impact on enterprises in regions with a higher level of marketization.

**Table 16:** Threshold regression test of marketization level.

Threshold variable	Threshold type	F-value	P-value	Crit-value			Threshold value
				1%	5%	10%	
Market	Single Threshold	12.66	0.000	5.848	7.258	12.381	
	Double Threshold	8.12	0.067	5.920	9.846	11.976	9.0060, 10.1530

**Table 17:** Double threshold regression result of marketization level.

Variable	Digital
AR(Market<9.01)	0.1401**
	(2.0323)
AR(9.01≤Market<10.15)	0.1924***
	(2.8997)
AR(Market≥10.15)	0.2379***
	(3.5170)
Age	-0.0388***
	(-7.0434)
Size	0.0136***
	(10.4719)
Roa	-0.0050
	(-1.3141)
Lev	-0.0123***
	(-5.5079)
Fix	0.0087
	(1.4425)
Shr	0.0017
	(0.2171)
Growth	0.0000
	(0.0974)
Cashflow	-0.0006
	(-0.2101)
RD	0.0446***
	(5.4216)
Urban	0.1444***
	(3.7501)
GDP	-0.0247***
	(-4.1313)
Constant	0.0040
	(0.0529)
Observations	28,874
AdjR-squared	0.0731

## 8. Conclusions and recommendations

### 8.1 Conclusions

This paper explores the impact and mechanisms of population aging on SME digital transformation. The study finds that, first, population aging has a significant positive impact on SME digital transformation and the result is robust to robustness checks and endogeneity tests. Second, the impact of population aging on SME digital transformation shows heterogeneity. For private enterprises and enterprises in the eastern regions of China, population aging can positively impact enterprise digital transformation; meanwhile, for state-owned enterprises and enterprises in the central-western regions of China, this impact is not significant. Third, regarding the influence mechanisms, population aging forces enterprises to replace labor with capital and technology and drives SMEs’ digital transformation; population aging facilitates the accumulation of human capital and provides intellectual support for SMEs’ digital transformation; population aging raises savings rates, alleviates financial constraints, and provides financial support for SMEs’ digital transformation. Fourth, the impact of population aging on SME digital transformation is dynamic and is influenced by both the degree of aging and the level of marketization. As the degree of population aging increases, the marginal positive impact of population aging on SME digital transformation shows a decreasing trend. As the marketization level improves, the impact of population aging on enterprise digital transformation gradually increases. This indicates that with the deepening of population aging, only by continuously improving the marketization level can the positive impact of population aging be attained.

### 8.2 Recommendations

Based on the research conclusions, this paper makes the following recommendations:

Improve the fertility security policy and plan a delayed retirement policy. Deepening population aging coupled with declines in the fertility rate will have a negative effect on labor in China. The state should support women’s reproductive rights at all levels of society. Tax incentives or subsidies to enterprises could reduce employment discrimination against women. The government should lower the threshold for maternity insurance, expand the coverage of maternity insurance, reduce fertility costs by rewarding multiple births, and increase women’s reproductive willingness. At the same time, the government should delay the legal retirement age to leverage the experience of elderly workers.

Improve public education quality and facilitate human capital accumulation. The state should continue to expand the coverage years and scope of compulsory education beyond the nine-year compulsory education. The government should support quality vocational education beyond guaranteeing a basic level of education for people. Highly skilled talent is necessary for leveraging innovative technologies after successful research and development. In addition, the government should increase investment in public education, reduce the cost of human capital accumulation, and enhance social education willingness.

Improve technological innovation ability and accelerate digital transformation. The state should increase investment in digital innovation and provide guaranteed technical support. The government should optimize relevant policies, guide resources toward enterprises with slower transformation speeds, and appeal to backward regions to raise their awareness of digital transformation. Enterprises should introduce digital technologies, reduce their reliance on low-end labor, and enhance their market competitiveness. Enterprises should also develop highly skilled employees, realize synergy between technology and capital factors, improve production efficiency, and enhance enterprise performance.

Strengthen marketization reform efforts and coordinate regional development levels. The state should prioritize speeding up marketization processes in the central-western regions of China, improving the allocation efficiency of production factors, enhancing market intermediary organizations, and optimizing the legal system environment. For eastern regions, the state should reduce unnecessary government intervention and allow market forces to freely operate to achieve equitable development among different regions.

## Supporting information

S1 Appendix Digital transformation keywords. (DOCX)

S1 Data Sharing data. (ZIP)
