# Supplementary material for: The impact of population aging on SME digital transformation: Evidence from China
Source: PLoS One. 2024 May 16;19(5):e0300660. doi: 10.1371/journal.pone.0300660 (PMC11098399; doi:10.1371/journal.pone.0300660)
Supplement: S1 Appendix — (DOCX) [file pone.0300660.s001.docx]

Appendix A: digital transformation keywords

| Digital Technologies | Artificial Intelligence | Artificial Intelligence (AI), Business Intelligence (BI), Image Understanding, Investment Decision Support System, Intelligent Data Analysis, Intelligent Robots, Machine Learning (ML), Deep Learning, Semantic Search, Biometric Technology, Facial Recognition, Speech Recognition, Identity Verification, Autonomous Driving, Natural Language Processing (NLP), Perceptual Interaction, Pervasive Perception |
| --- | --- | --- |
|  | Big Data | Big Data, Data Mining, Text Mining, Data Visualization, Heterogeneous Data, Credit Reporting, Augmented Reality, Mixed Reality, Virtual Reality, Visualization Algorithm |
|  | Cloud Computing | Cloud Computing, Stream Computing, Graph Computing, In-Memory Computing, Multi-Party Secure Computing, Brain-inspired Computing, Green Computing, Cognitive Computing, Converged Architecture, Billion-Level Concurrency, EB-Level Storage, Internet of Things (IoT), Cyber-Physical Systems, Edge Computing, Hybrid Cloud, Cloud Services, Cloud OS, Distributed Storage, Cloud IT, Cloud Platform |
|  | Blockchain | Blockchain, Distributed Computing, Differential Privacy Techniques, Smart Financial Contracts |
| Digital Application | Internet Business Model | Industry Internet, Internet Solutions, Internet Technology, Internet Thinking, Internet Action, Internet Business, Internet Mobile, Internet Application, Internet Marketing, Internet Strategy, Internet Platform, Internet Mode, Internet Ecology, E-business, Internet, "Internet+", Online to Offline, Online and Offline, Online, Offline, Mobile Internet, Industrial Internet, Mobile Interconnection, Internet Healthcare, E-commerce, Business-to-Business (B2B), Business-to-Consumer (B2C), Consumer-to-Business (C2B), Consumer-to-Consumer (C2C), Online-to-Offline (O2O), Network Alliance |
|  | Industrial Digitalization and Intelligence | High-end Intelligence, Industrial Intelligence, Mobile Intelligence, Intelligent Control, Intelligent Terminal, Intelligent Mobile, Intelligent Management, Intelligent Factory, Intelligent Logistics, Intelligent Warehousing, Intelligent Equipment, Intelligent Production, Intelligent Network, Intelligent System, Smart, Automatic Control, Automatic Monitoring, Automatic Supervision , Automatic Detection, Automatic Production, Numerical Control, Synthesis, Integration, Integration Solution, Integral Control, Integral System, Industrial Cloud, Future Factory, Intelligent Fault Diagnosis, Life Cycle Management, Manufacturing Execution System, Virtualization, Virtual Manufacturing, Smart Wearables, Smart Agriculture, Intelligent Transportation, Intelligent Healthcare, Intelligent Customer Service, Smart Home, Smart Investment, Smart Tourism, Smart Environmental Protection, Smart Grid, Smart Marketing, Digital Marketing, Unmanned Retail, Intelligent Energy, Smart Community, Smart Elderly Care, Smart Logistics, Smart Manufacturing, Smart Education, Smart Government, Digital Intelligence, Digital Terminal, Data Management, Data Network, Data Platform, Data Center, Data Science, Digital Control, Digital Technology, Digitalization |
|  | Digital Financial Service | Internet Finance, Digital Finance, Fintech, Financial Technology, Technology Finance, Quantitative Finance, Open Banking, Digital Currency, Mobile Payment, Third-party Payment, NFC Payment |
|  | Modern Information System | Digital Communication, Information Sharing, Information Management, Information Integration, Information Software, Information System, Information Network, Information Terminal, Information Center, Informatization, Networked, Industrial Information, Industrial Communication |
